# The Antiviral Effect of Novel Steroidal Derivatives on Flaviviruses

**DOI:** 10.3389/fmicb.2021.727236

**Published:** 2021-10-06

**Authors:** Luping Zhang, Dengyuan Zhou, Qiuyan Li, Shuo Zhu, Muhammad Imran, Hongyu Duan, Shengbo Cao, Shaoyong Ke, Jing Ye

**Affiliations:** ^1^State Key Laboratory of Agricultural Microbiology, Huazhong Agricultural University, Wuhan, China; ^2^Laboratory of Animal Virology, College of Veterinary Medicine, Huazhong Agricultural University, Wuhan, China; ^3^The Cooperative Innovation Center for Sustainable Pig Production, Huazhong Agricultural University, Wuhan, China; ^4^National Biopesticide Engineering Research Center, Hubei Biopesticide Engineering Research Center, Hubei Academy of Agricultural Sciences, Wuhan, China

**Keywords:** antiviral activity, flavivirus, steroids, DHEA derivatives, therapy

## Abstract

Flaviviruses are the major emerging arthropod-borne pathogens globally. However, there is still no practical anti-flavivirus approach. Therefore, existing and emerging flaviviruses desperately need active broad-spectrum drugs. In the present study, the antiviral effect of steroidal dehydroepiandrosterone (DHEA) and 23 synthetic derivatives against flaviviruses such as Japanese encephalitis virus (JEV), Zika virus (ZIKV), and Dengue virus (DENV) were appraised by examining the characteristics of virus infection both *in vitro* and *in vivo*. Our results revealed that AV1003, AV1004 and AV1017 were the most potent inhibitors of flavivirus propagation in cells. They mainly suppress the viral infection in the post-invasion stage in a dose-dependent manner. Furthermore, orally administered compound AV1004 protected mice from lethal JEV infection by increasing the survival rate and reducing the viral load in the brain of infected mice. These results indicate that the compound AV1004 might be a potential therapeutic drug against JEV infection. These DHEA derivatives may provide lead scaffolds for further design and synthesis of potential anti-flavivirus potential drugs.

## Introduction

Flaviviruses belonging to the family *Flaviviridae* are a group of more than 70 enveloped RNA viruses. They cause severe diseases in humans and animals. Most of them are arthropod-borne viruses (arboviruses) transmitted to humans or other vertebrate hosts by insect vectors (Gubler, 2007; Fernandez-Garcia et al., 2009). The Flavivirus genus including Japanese encephalitis virus (JEV), Zika virus (ZIKV), Dengue virus (DENV), West Nile virus (WNV), tick-borne encephalitis virus (TBEV) and yellow fever virus (YFV) (Mukhopadhyay et al., 2005). Some flaviviruses can cause encephalitis and other neurological manifestations, including WNV and JEV, while some tend to cause vascular leakage and hemorrhagic disease, including DENV and YFV (King et al., 2012; Barrows et al., 2018). Moreover, ZIKV has also been proved to be related to the development of Guillain–Barré syndrome in adults and severe fetal microcephaly (Culshaw et al., 2018).

Flavivirus infection is a global threat and is an endemic or epidemic in almost every tropical country. Flavivirus outbreaks still occur even the morbidity and mortality declined by using various vaccines, highlighting challenges in executing effective ways to control the epidemic (Campbell et al., 2011). To date, no specific commercial drugs against flaviviruses are available, so there is an urgent unmet need for new agents that can be used for prophylactic or therapeutic intervention in combating flavivirus infections. Recently, relevant kinds of literature have reported several compounds which have anti-flavivirus activities, such as ivermectin (Kok, 2016), manidipine (Wang et al., 2017), and chlorpromazine (Nawa et al., 2003). These studies underscore the value of developing novel uses for existing drugs, in addition to the discovery of new inhibitors against diseases caused by flavivirus infections.

Dehydroepiandrosterone (3β-hydroxyandrost-5-en-17-one, DHEA), a precursor of sex steroids, is one of the most plenteous steroids in human blood, a precursor of sex steroids (Hayashi et al., 2000). The peripheral levels of DHEA gradually decline with age (Barrett-Connor et al., 1986). The concentration of DHEA is exceptionally high in the brain tissue, since the neurons and astrocytes can synthesize DHEA (Zwain and Yen, 1999). DHEA has been proved to have chemoprotective effects against a series of diseases: diabetes, obesity, atherosclerosis, and cancer (Regelson et al., 2019). In addition, DHEA related hormones have been proved to exert antiviral effects on JEV, influenza virus, herpes simplex virus (HSV), vesicular stomatitis virus (VSV), human immunodeficiency virus (HIV), and Junin virus (JUNV) (Loria et al., 1988; Mulder et al., 1992; Chang et al., 2005; Acosta et al., 2008; Barrows et al., 2018). Therefore, the assessment of the antiviral activity of DHEA structurally related compounds can be used to pursue the effective treatment of viral infections. Many analogs have experimented with as antiviral agents *in vitro* and *in vivo* (Diallo et al., 2000; Torres et al., 2012).

**FIGURE 1:**
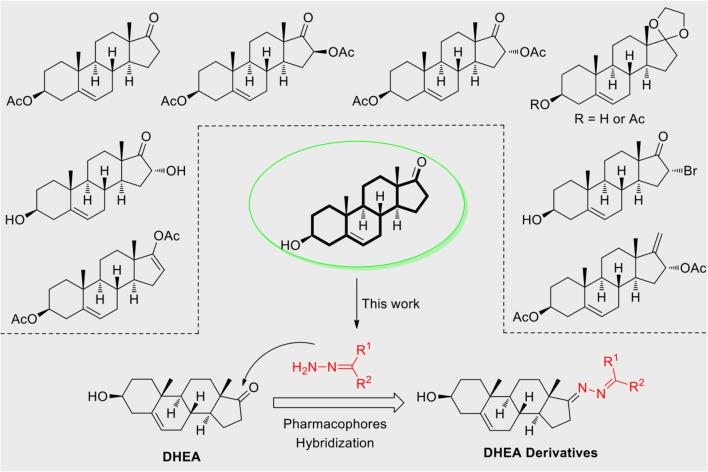
Derivatives design based on DHEA scaffold.

Based on the highly potential antiviral activity of DHEA derivatives (Acosta et al., 2008; Romanutti et al., 2009; Torres et al., 2012), a series of novel steroidal derivatives were constructed using pharmacophore hybridization, and it was supposed that introduction of dihydrazone active unit would increase the interaction sites of steroidal molecules (Figure 1). Moreover, assess whether the structural changes of DHEA would lead to improving antiviral properties, the antiviral activity of a series of steroidal derivatives based on DHEA scaffold against JEV, ZIKV, and DENV type 2 (DENV-2) infections both *in vitro* and *in vivo* was thoroughly investigated. The identified drugs in this study provide novel potential treatment protocols for flavivirus diseases.

## Materials and Methods

### Cell Cultures and Viruses

African green monkey kidney (Vero), Helen Lane (Hela), baby hamster kidney (BHK-21), and Human neuroblastoma (SHSY-5Y) cells were cultured and maintained in Dulbecco modified Eagle medium (DMEM; Sigma) supplement with 10% fetal bovine serum (FBS; Gibco), 100 U/ml penicillin, and 100 mg/ml streptomycin sulfate at 37°C in the 5% CO2. The JEV P3 strain was stored in our laboratory. China Center for Type Culture Collection (CCTCC) kindly provided the DENV type-2 strain and ZIKV H/PF/2013 strain.

### Chemicals and Reagents

All starting materials and reagents were supplied from commercial suppliers and used without further purification unless otherwise specified. All target steroidal molecules were efficiently synthesized in our laboratory according to the procedures described in our previous report (Ke et al., 2013). The physicochemical properties and the structures were elucidated by their corresponding NMR and ESI-MS comparison with the data in the literature. The substituents and structural formulae of target steroidal derivatives are indicated in Table 1. A stock solution was prepared in dimethyl sulfoxide (DMSO) with a concentration of 10 mM for storage and usage.

**TABLE 1:** Synthetic analogs of DHEA.

Sample No.	Compd. No.	R^1^	*R* ^2^	Structure
1	HAAS-AV1001	Ph	H	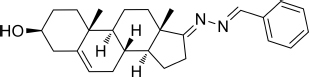
2	HAAS-AV1002	2-F-Ph	H	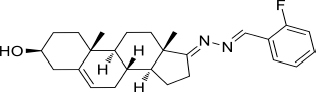
3	HAAS-AV1003	4-F-Ph	H	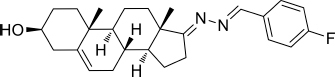
4	HAAS-AV1004	4-Cl-Ph	H	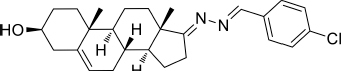
5	HAAS-AV1005	2,6-F_2_-Ph	H	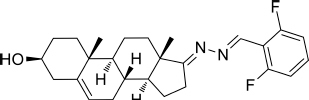
6	HAAS-AV1006	2,6-Cl_2_-Ph	H	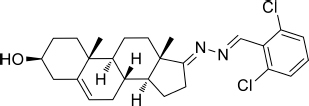
7	HAAS-AV1007	2,4-Cl_2_-Ph	H	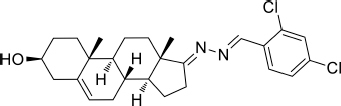
8	HAAS-AV1008	4-Br-Ph	H	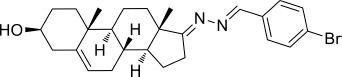
9	HAAS-AV1009	2-CN-Ph	H	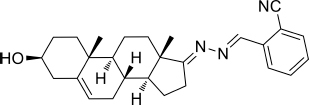
10	HAAS-AV1010	4-CF_3_-Ph	H	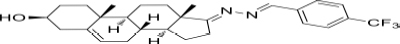
11	HAAS-AV1011	2-Cl-Ph	H	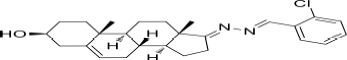
12	HAAS-AV1012	4-MeO-Ph	H	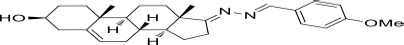
13	HAAS-AV1013	3,4,5-(MeO)_3_-Ph	H	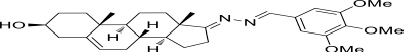
14	HAAS-AV1014	2-OH-Ph	H	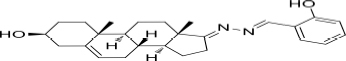
15	HAAS-AV1015	2-Py	H	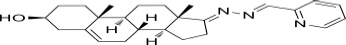
16	HAAS-AV1016	4-Py	H	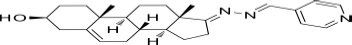
17	HAAS-AV1017	Indole-3-yl	H	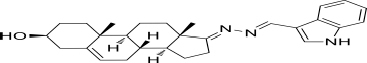
18	HAAS-AV1018	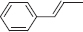	H	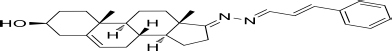
19	HAAS-AV1019	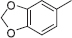	H	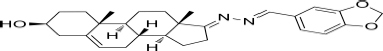
20	HAAS-AV1020	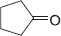		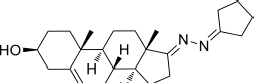
21	HAAS-AV1021	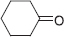		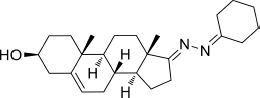
22	HAAS-AV1022	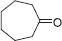		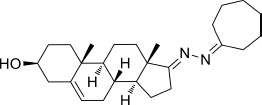
23	HAAS-AV1023	4-MeS-Ph	H	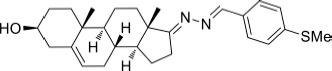
24	HAAS-AV1024	DHEA		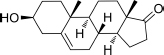

### Screening of Potential Anti-flavivirus Compounds

A series of DHEA derivatives were stored as 10 mM stock solutions in DMSO for storage and usage. Cells were infected with JEV (1 MOI). One hour later, the supernatant was removed, and the 10 μM DHEA derivatives or DMSO were added to the cells for additional 23 h. The antiviral effects of DHEA derivatives were determined by plaque assay. The compounds showing an inhibition ratio of > 90% were considered as candidates of subsequent experiments. The percentage of viral titer inhibition is the viral titer of the DMSO + JEV group minus the viral titer of the derivatives + JEV group and then divided by the viral titer of the DMSO + JEV group.

### Japanese Encephalitis Virus Infection and Drug Administration

Adult 6-week-old C57BL/6 mice were purchased from the Laboratory Animal Center of Huazhong Agricultural University, Wuhan, China. Mice were randomly assigned into 4 groups: control group (DMSO; *n* = 10); only AV1004-treated group (AV1004; *n* = 10); JEV and DMSO-treated group (JEV; *n* = 10); JEV- and AV1004-treated group (JEV + AV1004; *n* = 10). Mice belonging to the JEV + DMSO and JEV + AV1004 groups were intraperitoneally injected with 10^5^ PFU of the JEV P3 strain in 100 μl of DMEM. The compound stock solution was prepared in DMSO with a concentration of 10 mM for usage. The infected mice were given oral compound AV1004 at 35 mg/kg of body weight or DMSO for 23 consecutive days. The experimental animal protocols were approved by The Scientific Ethics Committee of Huazhong Agriculture University (HZAUMO-2021-0003).

### Cell Viability Assay

Using the Cell Titer-Glo^®^ One Solution Assay kit (Promega) to detect the viability of cells. The quantification of luminescence signals was calculated based on the reported method (Chen et al., 2018).

### Plaque Assay

Cell supernatants or cell lysates harvested at 12, 24, 36, and 48 h, were serially diluted in DMEM, then inoculated onto monolayers cells at 37°C for 1.5 h. Subsequently, the supernatants were removed, the cells were washed three times with serum-free DMEM and incubated with sodium carboxymethyl cellulose (Sigma) containing medium supplemented with 3% FBS. The cells were fixed with 10% formaldehyde and stained with crystal violet solution on day 4 post-incubation. Count the visible plaques and calculate the virus titer.

### Immunofluorescence Assay

Vero cells were infected with JEV and treated with different concentrations of compounds or DMSO. Cells were fixed at multiple time points with 4% formaldehyde for 10 min then washed with PBS. Afterward, cells were blocked with 10% bovine serum albumin (BSA) in PBS for 1 h. Later, cells were incubated with JEV E mAb 4B4 for 3 h. After washing with PBS, cells were stained with a second antibody (Alexa Fluor 488) for 30 min. Cell nuclei were stained with DAPI. The staining results were observed under an fluorescence microscope (Zeiss).

### Western Blotting

Cells were harvested and incubated in lysis buffer (Beyotime Biotechnology, Shanghai, China) for 15 min with ice-cold water, then the supernatants were collected after centrifuging at 12,000 rpm for 10 min at 4°C. Using the BCA protein assay kit (Thermo Fisher Scientific, Waltham, MA, United States) to determine the protein concentration. Proteins were separated by SDS-PAGE and diverted to a nitrocellulose membrane using a Mini *Trans-*Blot Cell (Bio-Rad Laboratories). The membrane was incubated with the JEV E mAb 4B4 or JEV NS5 mAb 2A5 as requested, and signals were explored using ECL reagents (Thermo Fisher Scientific).

### RNA Extraction and Quantitative Real-Time PCR

Total cellular RNA was obtained using TRIzol Reagent (Invitrogen), and reverse transcription of RNA was performed with the ABscript II cDNA First Strand Synthesis kit (ABclonal). qRT-PCR was performed using SYBR Green First qPCR Mix (ABclonal) and the QuantStudio 6 Flex PCR system (Applied Biosystems). The primers are listed in Table 2.

**TABLE 2:** Primers used for qRT-PCR.

Primer Name	Sequence 5′-3′
m(β-actin-F	CACTGCCGCATCCTCTTCCTCCC
m(β-actin-R	CAATAGTGATGACCTGGCCGT
mIL-1(β-F	AACCTGCTGGTGTGTGACGTTC
mIL-1(β-R	CAGCACGAGGCTTTTTTGTTGT
mIL-6-F	AATGAGGAGACTTGCCTGGT
mIL-6-R	GCAGGAACTGGATCAGGACT
mCCL-2-F	CGGCGAGATCAGAACCTACAAC
mCCL-2-R	GGCACTGTCACACTGGTCACTC
mCCL-5-F	TGCCCACGTCAAGGAGTATTTC
mCCL-5-R	AACCCACTTCTTCTCTGGGTTG
hβ-actin-F	AGCGGGAAATCGTGCGTGAC
hβ-actin-R	GGAAGGAAGGCTGGAAGAGTG
JEV-F	GGCTCTTATCACGTTCTTCAAGTTT
JEV-R	TGCTTTCCATCGGCCYAAAA
ZIKV-F	TAAACGGGGTTGTCAGGCTC
ZIKV-R	ACCTGACGAGTGCCTTCTTG
DENV-F	CTGAAACGCGAGAGAAACCG
DENV-R	GTATCCCTGCTGTTGGTGGG

*h, human; m, mouse; F, Forward; R, Reverse.*

### Time-of-Addition Assay

To determine which stage of the JEV life cycle was inhibited by each treatment, BHK-21 cells were treated with JEV (1 MOI) for 1 h (0 to 1 h). The test compounds were incubated with the cells or virus for 1 h pre-infection (−1 to 0 h), co-infection (0 to 1 h), and post-infection (1 to 24 h). At 24 h post-infection, the titer of supernatant was determined by plaque assay. The cell lysates were immunoblotted to detect the viral protein expression level.

### Hematoxylin and Eosin Staining, Immunohistochemistry Assay

Ketamine-xylazine (0.1 ml per 10 g of body weight) was used to anesthesia the treated mice. Brain tissues were collected then embedded in paraffin for coronal sections. The sections were used for H&E staining and Immunohistochemistry (IHC) assay. For IHC, the antibodies ionized calcium-binding adapter molecule 1 (IBA-1) (Wako) and glial fibrillary acidic protein (GFAP) have been used in our previous studies (Chen et al., 2018). Using ImagePro Plus software to analyze the number of positive cells for each antibody to obtain the comprehensive option density index.

### Japanese Encephalitis Virus Replicon Assay

To ensure the compound’s effectiveness in JEV replication, the JEV replicon cDNA clone in which the structural genes were replaced with the Renilla luciferase (Rluc) gene was employed to quantitatively evaluate the inhibitory effects (Li et al., 2014). *In vitro* transcripts were synthesized from linearized JEV replicon using a T7 mMessage mMachine kit (Invitrogen). Transfection of *in vitro* transcripts into Hela cells. At the indicated times post-transfection, using the Rluc assay system (Promega) to measure the luciferase activity.

### Statistical Analysis

All experiments were performed at least three times under similar conditions. Analyses were conducted using GraphPad Prism Software (version 8). Results were expressed as the mean ± standard error of the mean (SEM). Statistical differences were determined using the two-way analysis of variance (ANOVA) with subsequent Student’s test, or the Student *t*-test, or the Log-rank (Mantel-Cox) test. *P* < 0.05.

## Results

### Screening of Potential Anti-flavivirus Compounds From Dehydroepiandrosterone and Its Derivatives

**FIGURE 2:**
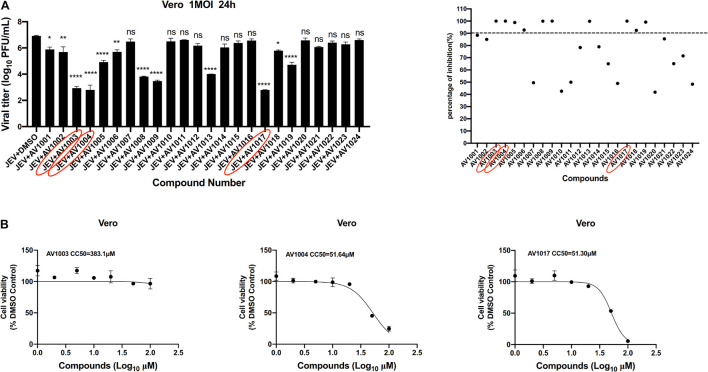
Screening of potential anti-flavivirus compounds from DHEA and its derivatives. **(A)** Vero cells were seeded at a density of 5 × 10^4^cells/well in 48-well plates. After 16 h incubation, the cells were treated with JEV (1 MOI). 1 h later, the supernatant was removed and the 10 μM DHEA derivatives were added to the cells for an additional 23 h. The antiviral effects of DHEA derivatives were determined by plaque assay. The criterion for screening compounds is the inhibition rate > 90%. Each dot represents the percent inhibition of each compound at a concentration of 10 μM. **(B)** Cell viability determination of DHEA derivatives at indicated concentrations in Vero cells. *P < 0.05, **P < 0.01, ****P < 0.0001.

The antiviral abilities of DHEA and its derivatives against JEV were examined by inhibition of virus-induced cytopathogenicity effects (CPEs) in BHK-21. Among the 23 molecules, we found that 10 derivatives inhibited JEV infection by more than 90%. However, the inhibition rate of DHEA (AV1024) on JEV replication was only 50%. This result was consistent with that reported in the previous article (Chang et al., 2005). And the three compounds, AV1003, AV1004, and AV1017, showed the strongest antiviral effects were selected for the following experiments (Figure 2A). The luminescence-based viability assay was used to evaluate the cytotoxic effects of the three derivatives in Vero cells. The 50% cytotoxic concentration (CC_50_) of derivatives, were identified as 383.1 μM (AV1003), 51.64 μM (AV1004) and 51.30 μM (AV1017), respectively (Figure 2B). The highest non-toxic concentration of AV1003, AV1004, and AV1017 (20 μM) was used for subsequent experiments.

### Dehydroepiandrosterone Derivatives Inhibit the Propagation of Japanese Encephalitis Virus *in vitro*

**FIGURE 3:**
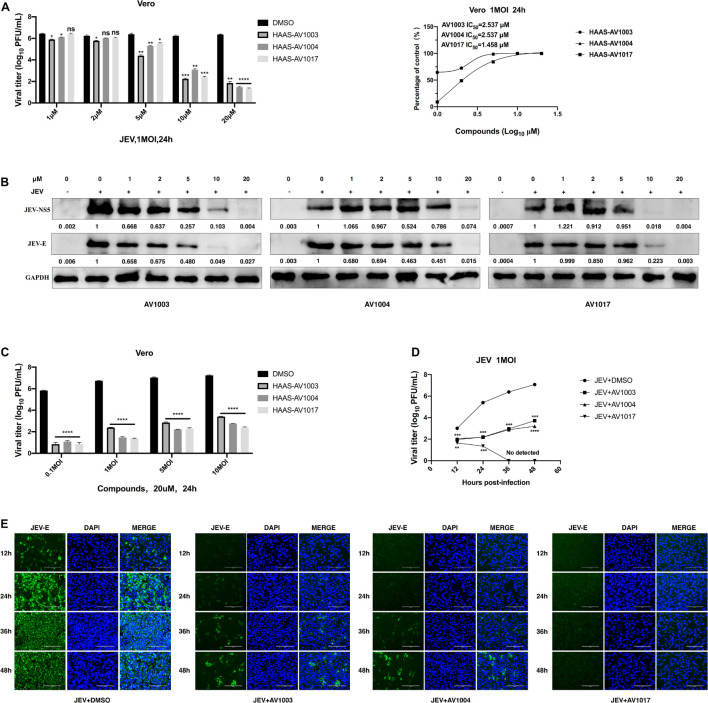
Evaluation of the antiviral effects of the DHEA derivatives against JEV. **(A,B)** Vero cells were treated with JEV (1 MOI) followed by treatment with different concentrations of DHEA derivatives. The viral titer in cell culture supernatants was determined by plaque **(A)** and the expression of viral proteins was determined by western blot analysis **(B)** at 24 h post-infection, the IC_50_ was calculated **(A)**. **(C)** Vero cells were treated with indicated MOI of JEV followed by treatment with 20 μM DHEA derivatives. At 24 h post-infection, the cell culture supernatants were determined by plaque assay. **(D,E)** The effect of derivatives on JEV replication. Vero cells were treated with JEV (1 MOI). After 1 h incubation, cells were treated with 20 μM DHEA derivatives. Viral titers **(D)** and JEV E protein expression **(E)** at indicated time points were measured by plaque assay and immunofluorescence analysis, respectively. *P < 0.05, **P < 0.01, ***P < 0.001, and ****P < 0.0001.

To verify the antiviral effect of AV1003, AV1004, and AV1017 against JEV, several *in vitro* experiments were carried out. The antiviral activity of AV1003, AV1004, and AV1017 against the flaviviruses was examined by plaque assay. The JEV infected Vero cells were immediately incubated in media containing different non-cytotoxic concentrations of DHEA derivatives. Virus yield was quantified at 24 h.p.i and compared to untreated virus-infected cultures. Virus multiplication was observed with a dose-dependent inhibition in all three derivatives-treated cultures by plaque assay and Western blot (Figures 3A,B). The 50% inhibitory concentration (IC_50_) of AV1003, AV1004, and AV1017 were determined to be 2.537, 2.537, and 1.458 μM against JEV, respectively (Figure 3A). The levels of viral proteins have been quantified and normalized to GAPDH by using Image J software (Figure 3B), which showed that the higher the derivative concentration, the lower the viral protein expression. The CC_50_ and IC_50_ values were used to derive the selectivity index (SI) value for each compound (Table 3). At the highest non-cytotoxic concentration, the compound’s inhibitory effect on different MOIs of virus and time points was determined. It was found that the three compounds still have strong antiviral activity even at a higher MOI of 10 (Figure 3C). As showed by plaque assay, the production of infectious viral particles in the compounds treated infected cells reduced more than 2-log at 24, 36, and 48 h post-infection (Figure 3D). Based on immunofluorescence analysis, the JEV-E protein expression was significant inhibited by the three compounds (Figure 3E). These results indicate that these compounds have a strong anti-JEV effect.

**TABLE 3:** CC_50_, IC_50_, and SI values of derivatives on Vero cells, derived from data obtained from the cell viability and dose-dependent inhibition assays, respectively.

Sample No.	Compd. No.	Structure	IC_50_(μM)	CC50(μM)	SI
3	HAAS-AV1003	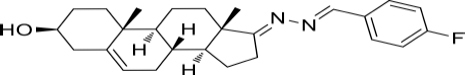	2.537	383.1	>151
4	HAAS-AV1004	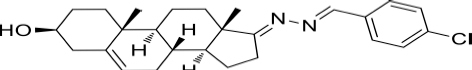	2.537	51.64	>20.35
17	HAAS-AV1017	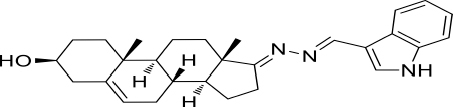	1.458	51.30	>35.19

### The Antiviral Effects of Dehydroepiandrosterone Derivatives Is Cell Type-Independent

**FIGURE 4:**
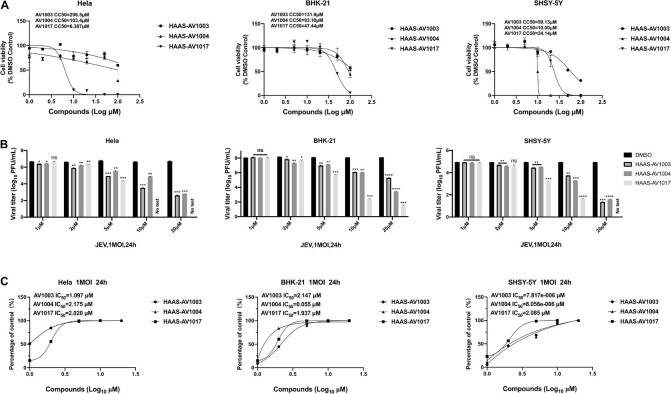
Validation of the antiviral effects of derivatives in different cell lines. **(A)** Cell viability assay at different concentrations of DHEA derivatives in Hela, BHK-21 and SHSY-5Y cells. **(B,C)** Hela, BHK-21 and SHSY-5Y cells were treated with JEV (1 MOI), followed by treatment with multiple concentrations of DHEA derivatives for 24 h. Viral titers **(B)** at indicated cell lines were measured by plaque assay, and the IC_50_ was calculated **(C)**, respectively. *P < 0.05, **P < 0.01, ***P < 0.001, and ****P < 0.0001.

To investigate whether the compound-induced anti-JEV effect is influenced by cell type, the effect of highest non-toxic concentration of compounds on JEV replication in Hela, BHK-21 and SHSY-5Y cells was examined (Figure 4). We examined the cytotoxicity of hits by luminescence-based cell viability assay. Among the three derivatives, compound AV1017 containing an indole unit showed the potent cytotoxicity for cell lines, so we detected the antiviral effect of 5 μM compounds on Hela cells and 10 μM compounds on SHSY-5Y cells. The results of different concentrations of each compound on the production of infectious progeny were assessed. At 24 h.p.i., supernatants of infected cell cultures were harvested and titrated by plaque assays. The dose-dependent inhibitory effect of AV1003, AV1004, and AV1017 was recapitulated in JEV-infected Hela, BHK-21 and SHSY-5Y cells (Figure 4B), albeit at a decreased potency relative to drug treatment on JEV-infected Vero cells.

### Dehydroepiandrosterone Derivatives Inhibit the Replication of Zika Virus and Dengue Virus *in vitro*

**FIGURE 5:**
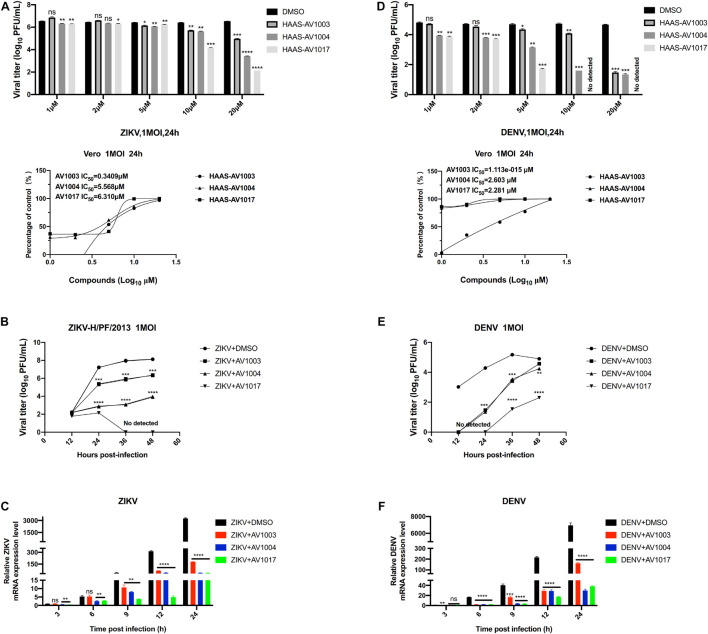
Broad-spectrum antiviral activity of the derivatives against flaviviruses. **(A,D)** Vero cells were treated with ZIKV **(A)** or DENV **(D)** at 1 MOI for 1 h. The indicated concentrations of derivatives were added to the cells at 1 h post-infection and were present throughout the whole experiment. At 24 h post-infection, the cell culture supernatants were determined by plaque (top panel), the IC_50_ was calculated (bottom panel), respectively. **(B,C,E,F)** Vero cells were treated at 1 MOI with either ZIKV or DENV. Subsequently, infected cells were treated with derivatives (20 μM) for 48 h. ZIKV **(B,C)** or DENV **(E,F)** titers and mRNA levels at the specified time point were quantified by plaque assay and qRT-PCR experiment, respectively. *P < 0.05, **P < 0.01, ***P < 0.001, ****P < 0.0001.

The antiviral ability of compounds against other flaviviruses such as DENV and ZIKV was investigated. Similar to JEV, DHEA derivatives significantly inhibit the replication of ZIKV (Figures 5A–C) and DENV (Figures 5D–F) in Vero cells at different time points. A drug-dose-dependent manner was proved by plaque and qRT-PCR assay. Among the three DHEA derivatives, AV1017 and AV1004 showed the most potent antiviral effect on ZIKV and DENV, respectively (Figures 5A,D). Subsequently, the antiviral efficacy of compounds against ZIKV (1 MOI) and DENV (1 MOI) in Vero cells was measured at multiple time points. According to Figure 5B, the derivatives exert an antiviral effect against ZIKV infection after 12 h. AV1004 and AV1017 have more substantial inhibitory effects on ZIKV. In Figure 5E, when DENV infected cells for 12 h, no virus particles were detected in the compound treatment groups, and the virus titer gradually increased over time. The results indicated that the replication of ZIKV and DENV was inhibited by compounds at indicated time points. The derivatives have a more substantial inhibitory effect on the early stage of DENV infection (Figures 5B,C,E,F). In addition, the infectivity of ZIKV and DENV on Vero cells were different, which may also be considered as a possible reason for affecting the early steps of DENV and ZIKV life cycle differentially. These data indicated that compounds exert broad-spectrum anti-flavivirus activity.

### Dehydroepiandrosterone Derivatives Inhibit Flavivirus Multiplication After Viral Entry and Before Genomic Replication

**FIGURE 6:**
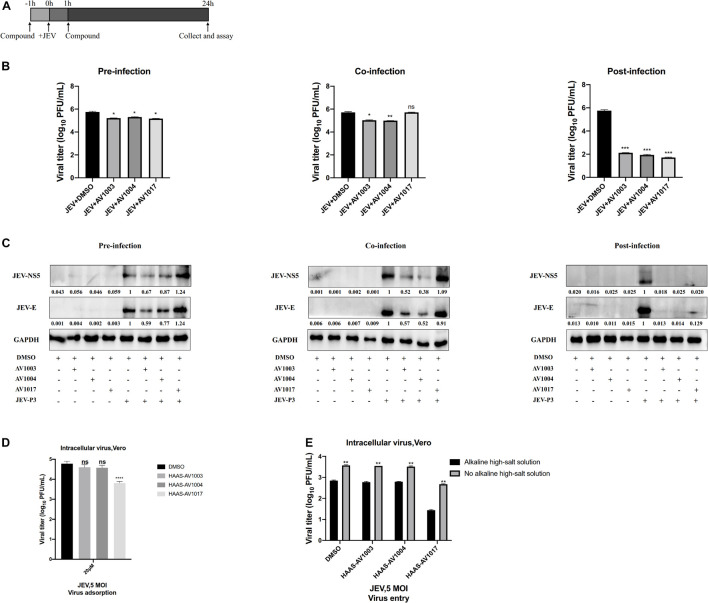
Time-of-addition analysis of the antiviral activity of the derivatives against JEV. **(A)** Brief schematic diagram of time-of-addition experiment. **(B,C)** Vero cells were treaed with JEV (1 MOI) for 1 h (0 to 1 h). AV1003 (10 μM), AV1004 (10 μM) and AV1017 (10 μM) were introduced at various time points of virus infection, pre-infection (pre), co-infection(co), or post-infection (post). The inhibitory effect of the drugs in each group was determined by plaque assay **(B)** and immunofluorescence analysis **(C)**. **(D)** Vero cells were treated with JEV (5 MOI) + AV1003/AV1004/AV1017 and incubated at 4°C for 1 h. Samples were collected to detect the virus titer by plaque assay. **(E)** Vero cells were treated with JEV (5 MOI) at 4°C for 1 h. Then cells were washed 3 times and treated with AV1003, AV1004 and AV1017 (10 μM) at 37°C for another 1 h. Samples were collected to detect the virus titer by plaque assay. *P < 0.05, **P < 0.01, ***P < 0.001, ****P < 0.0001.

To delineate at which stage in virus life cycle compounds work, whether compounds impede a pre-infection or post-entry stage was determined. Cultured Vero cells were subjected to a time-of-addition assay: pre-infection, co-infection and post-infection (Figure 6A). The levels of viral proteins quantification have been done by using Image J software (Figure 6C), which showed that treatment of compounds post-infection reduced the viral particles production and viral protein expression to the most significant degree than treatment of compounds pre-infection and co-infection. Nonetheless, according to the plaques and western blot results, these effects were found less significant upon pre-infection and co-infection of compounds with the virus (Figures 6B,C). These results indicated that the derivatives mainly exert an inhibitory effect after viral infection.

The effect of compounds on cellular adsorption and entry phases of the virus life cycle was verified. To detect the effect of compounds on the attachment step, after 1 h incubation with JEV (5 MOI) + DMSO or JEV (5 MOI) + AV1003, AV1004, and AV1017 at 4°C, the cells were washed with PBS to remove the unattached virus. The amounts of viruses attached to the cell surface were measured by plaque assay. No difference between JEV + AV1003/AV1004 and the JEV + DMSO group was shown. However, the virus titer of the JEV + AV1017 treatment group was reduced (Figure 6D). To detect the effect of compounds on the invasion step, cells were incubated with the virus at 4°C for 1 h, followed washed three times and treated with AV1003, AV1004, and AV1017 (10 μM) at 37°C for another 1 h to initiate viral entry. To remove the free virus and cell surface-associated virus, the infected cells were rigorously washed by alkaline high-salt solution. The titer of intracellular viral was quantified by plaque assay. Similar to the attachment results, viral titers of JEV + AV1003/AV1004 showed no significant difference, while JEV + AV1017 viral titer was lower than that of JEV + DMSO group viral (Figure 6E). These findings suggested that AV1003 and AV1004 do not disrupt the cellular adsorption and entry ability of the virus, but AV1017 affects both adsorption and invasion stages of the virus.

**FIGURE 7:**
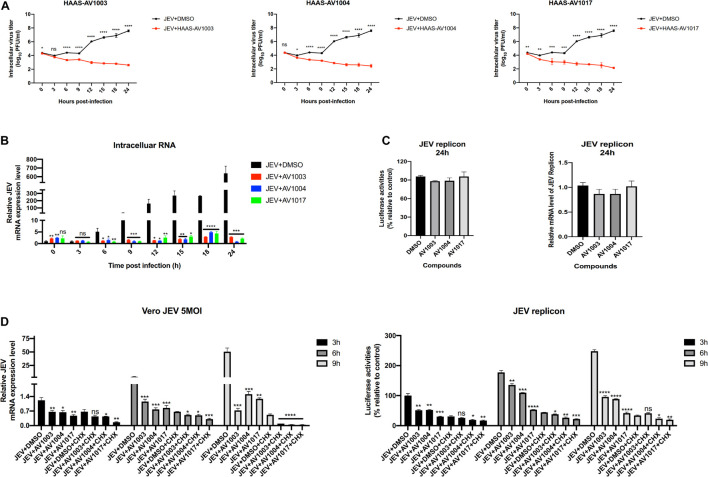
DHEA derivatives play an inhibitory role in the early stage of viral infection. **(A,B)** Vero cells were treated with JEV (5 MOI) for 1 h followed by derivatives (20 μM) or DMSO treatment. Cells were subjected to one freeze-thaw cycle to liberate cell-associated viruses at the indicated time points post-infection. Intracellular **(A)** virus titer was determined by plaque assay. And the JEV mRNAs levels **(B)** at specified time points were quantified by the qRT-PCR experiment. **(C)** Hela cells transfected with the JEV replicon were treated with derivatives at 10 μM, luciferase activities and mRNAs levels were determined at 24 h. **(D)** Vero cells were treated with JEV (5 MOI) or Hela cells transfected with the JEV replicon followed by treatment with 20 μM of DHEA derivatives and 100 μg/ml of CHX. The mRNAs levels and luciferase activities at specified time points were quantified. *P < 0.05, **P < 0.01, ***P < 0.001, and ****P < 0.0001.

To clarify at which stage of the virus infection the derivatives work, JEV-infected cell supernatants from the control (DMSO) and treatment (compounds 20 μM) groups were subjected to plaque assay and RT-qPCR at different time points. The plaque-forming unit (Figure 7A) and intracellular mRNA levels (Figure 7B) showed significant differences between the control and treatment groups. To determine whether the derivatives could inhibit flavivirus genomic replication, Hela cells were transfected with the JEV replicon followed by treatment with the derivatives. However, there was no significant change in the luciferase signal and mRNA levels at 24 h post-transfection (Figure 7C), suggesting that the derivatives could not inhibit the genomic replication of JEV. These results indicated that the antiviral activity of derivatives against JEV infection occurs after viral entry and before genomic replication.

Cycloheximide (CHX) is a compound that has an inhibitory effect on protein biosynthesis in eukaryotes, and it is a product of Streptomyces griseus. It hinders the translation process by interfering with the translocation step in the protein synthesis process. In biopharmaceutical research, cycloheximide is often used to inhibit protein synthesis in eukaryotic cells *in vitro* (Ramanathan et al., 2020). Afterward, an additional time course assay was performed with JEV and three derivatives in the presence or absence of CHX. Besides, we performed the cycloheximide experiment using the JEV replication system. Vero cells were treated with JEV (Figure 7D left panel) or Hela cells were transfected with the JEV replicon (Figure 7D right panel), and treated with the derivatives or the derivatives + CHX. Upon treatment of CHX, the RNA levels and the luciferase signal of the JEV + AV1003, AV1004, and AV1017 groups were still lower than that of the DMSO group (Figure 7D). Taken together, these results demonstrated that the three compounds play the antiviral role against JEV infection after virus entry and before genomic translation.

### AV1004 Reduce the Lethality of Japanese Encephalitis Virus Infected Mice

**FIGURE 8:**
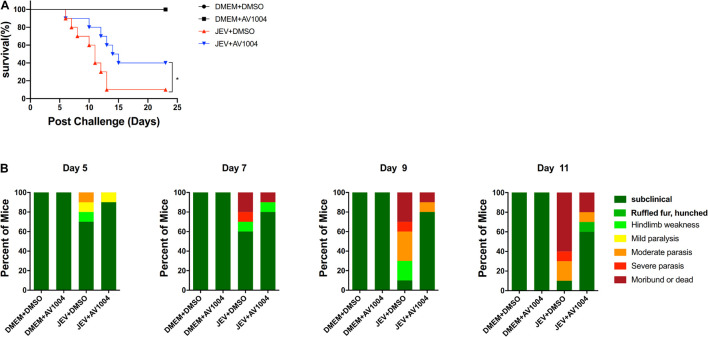
AV1004 protects mice from JEV infection. **(A)** Monitoring the survival rate of mice in each group for 23 days after inoculation with JEV. *n* = 10 mice per group. **(B)** The clinical symptoms of the disease in mice are demonstrated on indicated days post-infection. *n* = 10 mice per group. Mean values were considered significantly different using the Log-rank (Mantel-Cox) test. *P < 0.05.

Given the best performance of AV1004 on inhibiting JEV, ZIKV and DENV propagation, it was selected to evaluate the therapeutic effect on JEV infected mice. JEV- or mock-infected mice were treated with AV1004 or DMSO at 35 mg/kg of body weight for 23 consecutive days. The *in vivo* results showed that all mice survived in the AV1004- or DMSO-treated group throughout the entire observation period. JEV-infected mice treated with DMSO or AV1004, mortality displayed on day 6 post-infection. On day 13 post-infection, the mortality rate was 90% in the DMSO-treated mice. In contrast, the survival rate of AV1004 protected mice was 40% throughout the entire 23-day period of observation (Figure 8A). To substantiate the effect of AV1004 on clinical symptoms, we scored the behavioral signs of mice in all the experimental groups. Compared to the DMSO-treated group, the AV1004-treated group improved the behavioral signs of JEV-infected mice. No obvious clinical symptoms were found of non-infected mice in AV1004 and DMSO control groups during the observation period (Figure 8B). These results indicated that the AV1004 could reduce mortality caused by JEV infection.

### AV1004 Reduce the Viral Load and Inflammatory Response in Brain Tissue of Japanese Encephalitis Virus Infected Mice

**FIGURE 9:**
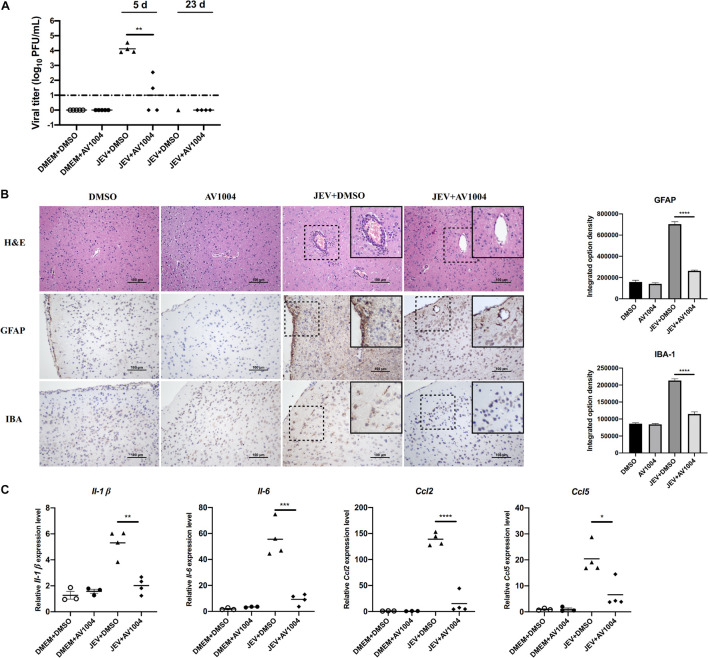
AV1004 reduces the JEV-induced neuroinflammatory response *in vivo*. **(A)** Viral titers in the mouse brain tissue on days 5 and 23 post-infection were determined by plaque assay. Each data point represents one mouse. **(B)** IHC and H&E staining to observe the activation of glial cells and pathological changes. Black box visualization indicates activated glial cells and perivascular cuffing. Scale bar, 100 μm. Integrated option density analysis was performed to quantify the results of immunohistochemical staining. Data represent mean ± SEM of 3 sections from 3 mice from each group. **(C)** The mRNA expression levels of inflammatory cytokines (*Il-1*β, *Il-6*, *Ccl2* and *Ccl5*) in brain tissue lysates were quantified by qRT-PCR. Each data point represents one mouse. *P < 0.05, **P < 0.01, ***P < 0.001, and ****P < 0.0001.

Mice were sacrificed on days 5 and 23 post-infection. Tissue samples were collected and processed for the subsequent experiments. The viral loads in infected mice brain tissues were measured by plaque assay. Compared to the JEV-infected + DMSO-treated group, brain homogenates prepared on day 5 post-infection from JEV-infected + AV1004-treated mice, viral titers were reduced significantly (Figure 9A). On day 23 post-infection, all the survivals were recovered, no virus was detected in those mice. To assess the role of A1004 in attenuating the JEV induced massive inflammatory response in brain tissue. H&E, IHC staining and quantification of proinflammatory cytokines analysis were conducted on day 5 post-infection collected brain samples. The histopathological changes of the cerebrum in JEV-infected mice showed perivascular cuffing and meningitis, but these indicators of encephalitis were declined in JEV-infected + AV1004 treated mice (Figure 9B). To investigate the effect of AV1004 treatment on JEV-induced gliosis, IHC staining was performed by using the antibodies which can recognize the microglia marker protein IBA-1 and the astrocyte marker protein GFAP. The results suggested that AV1004 reduces reactive astrogliosis and microgliosis in infected mice. Furthermore, the expression of proinflammatory cytokines, such as *Il-1* β, *Il-6*, *Ccl2*, and *Ccl5* were measured by RT-qPCR assay. A significant reduction of RNA levels of proinflammatory cytokines was observed upon treatment of AV1004 was observed (Figure 9C). These results indicate that A1004 has a potential therapeutic effect on reducing inflammation mediated by JEV.

## Discussion

The annual incidence of flavivirus infections in humans has reached millions of cases, no specific anti-flavivirus therapy has been developed yet (Campbell et al., 2011; Diamond and Pierson, 2015; Weaver et al., 2016). The increasing flavivirus infections worldwide highlight the urgent need for novel antiviral drugs. In accordance with the analysis of chemical structures of known drugs, it becomes feasible to design and synthesize novel drug derivatives, making the starting point of modern drug discovery. Considering the low cost, short cycles, and high benefits, the transformation of natural products is conducive to the construction of a significant number of molecular libraries for activity screening, thereby facilitating the development of high potential antiviral agents.

Dehydroepiandrosterone (DHEA) is an intermediate in testosterone biosynthesis and a typical steroid and sex hormone precursor, which can affect the formation of fat, regulate NADPH, interfere with the production of interleukins and interferons, and bind endothelial cell membrane receptors (Berdanier et al., 1993; Labrie et al., 2003). DHEA has been characterized as an antiviral compound by previous studies as well as a potential therapeutic drug for specific viruses, for instance, HIV, JUNV, HSV, HCV, etc. (Loria et al., 1988; Mulder et al., 1992; Acosta et al., 2008; Barrows et al., 2018). To discover novel molecules as potential antiviral agents against flaviviruses, a series of steroidal derivatives based on DHEA scaffold were designed and synthesized in the present study. With the DHEA scaffold serving as the core structure, various aryl-hydrazone pharmacophores were integrated into the steroidal structure from the perspective of structure-activity relationships. The transformation was carried out at 17 positions of DHEA, with the resulting steroidal derivatives displaying more vital anti-flavivirus ability in comparison to that of DHEA. Therefore, it is of great practical significance to introduce this series of compounds as potential flavivirus inhibitors. We accordingly examined the antiviral and cytotoxic effects of DHEA and 23 synthetic derivatives using Vero cells. In this study, we found three-hit compounds and SI index greater than 30, which exert inhibitory effects on flavivirus. Among the three derivatives, compounds AV1003 and AV1004 were less toxic to different cells, with CC_50_ values higher than 100 μM.

The time-of-drug additional assay revealed that AV1003, AV1004, and AV1017 worked more efficiently at post-infection. The effectiveness of AV1003, AV1004, and AV1017 against flavivirus infections was determined, respectively, at different time points (24, 36, and 48 h.p.i.) according to their ability to lower viral titers and impede virus spread from infected cells to neighboring cells. During a flavivirus life cycle, the viral particle is internalized by receptor-mediated endocytosis, and upon endosome acidification, conformational changes in the E protein leads to membrane fusion (Modis et al., 2004; Nayak et al., 2009). The early stages of the flavivirus life cycle include binding, entry into the cells, uncoating, translation, and RNA replication (Ramanathan et al., 2020). Among the three derivatives, AV1017 affects both adsorption and invasion stages of the virus in Vero cells. The AV1017 provides a foundation for the development of JEV entry inhibitors. Recently, some relevant researches about inhibitors of JEV entry were reported. Berbamine, a bisbenzylisoquinoline alkaloid that is isolated from herbs. It’s an effective anti-JEV agent, acting as a calcium channel or signaling inhibitor to prevent JEV entry (Huang et al., 2021). The sulfated polysaccharides “Carrageenan,” a linear sulfated polysaccharide is extracted from red edible seaweeds, inhibits the early stages of JEV infection (Nor Rashid et al., 2020). As known that each replication cycle of flavivirus takes about 16 h (Mukherjee et al., 2011), our study suggests that AV1003/1004 can inhibit virus propagation at early steps after viral entry and before genomic replication. There are multiple endocytic pathways for JEV to be internalized from the host cell’s plasma membrane to the endosomal compartment, which largely depends on the type of infected cells (Mayor and Pagano, 2007). The classical clathrin-dependent pathway was observed in BHK-21 (Liu et al., 2017), Vero (Nawa et al., 2003; Kalia et al., 2013), and PK15 (Yang et al., 2013). The non-classical clathrin-independent pathway was observed in the human neuroblastoma SK-N-SH (Xu et al., 2016), and mouse neuroblastoma Neuro-2a (Kalia et al., 2013). Considering the variations mentioned above, it is essential to investigate the main endocytic pathway for JEV to enter human brain neurons, peripheral human monocytes and macrophages/DCs, which may play an essential role in neuroinvasion.

However, the molecular mechanism by which DHEA derivatives inhibit viruses remains unclear. The observed antiviral action of these derivatives may be associated with the host-signaling machinery. Therefore, the host factors had been already known to facilitate viral replication will provide clues for identifying the targets of those compounds. Recently, several reports suggest the role of a host signaling molecule that plays a vital role in virus replication post-invasion and at the time of genome replication. For instance, research proves that the targeted inhibitory effect of the virus on Dihydroorotate dehydrogenase (DHODH) can block the synthesis of new pyrimidines and cause DNA synthesis obstacles, thereby effectively inhibiting the transcription and replication of viral RNA in host cells (Liu et al., 2000). The ubiquitin-proteasome system (UPS) was also involved in JEV entry, especially in the post-attachment step, which was before the initial translation of viral genomic RNA (Wang et al., 2016). The virus-host cell membrane fusion is a unique event in several flaviviruses (JEV and YFV) which was proved by the biochemical assays combined with live-cell imaging and single-particle tracking. The fusion precedes the microtubule-mediated viral nucleocapsid/genome released into the cytoplasm (Nour et al., 2013). Besides, a recent study demonstrated that during the entry of DENV, the viral genome releasing or nucleocapsid uncoating is hampered by inhibiting ubiquitination (Byk et al., 2016). Transitional endoplasmic reticulum 94 (TER94) plays an essential role in viral uncoating by interacting with ZIKV capsid and trafficking it for proteasomal degradation (Gestuveo et al., 2021). Therefore, further research is needed to determine the exact role of DHEA derivatives post-entry of JEV and other flaviviruses in the host cells.

Meanwhile, the efficacy and toxicity of AV1004 were monitored in mice. AV1004 showed effective antiviral effect with favorable biocompatibility. It was observed that treated with AV1004 inhibited viral replication in JEV infected mice as well as alleviated manifestations of encephalitis in mouse brain tissues. This offers strong evidence that AV1004 is a potential therapeutic drug against JEV infection. The revamp of the DHEA structure also offers new insights for developing effective drugs against viral infections.

In conclusion, our *in vitro* and *in vivo* experimental findings demonstrate that DHEA derivatives could inhibit flaviviruses propagation. AV1004 exhibits significant antiviral activity against JEV infection within the period between viral entry and genomic replication. AV1004 protects mice from JEV-induced lethality and attenuates the histological manifestations of JEV through reduced viral loads and histopathological damage in the brain tissue. AV1004 is a potential treatment option for inhibiting multiple flaviviruses infections. In addition, this study suggests DHEA derivatives may provide a promising molecule framework basis for the further design and synthesis of potential antiviral drugs.

## Data Availability Statement

The original contributions presented in the study are included in the article/supplementary material, further inquiries can be directed to the corresponding author/s.

## Ethics Statement

The animal study was reviewed and approved by The Scientific Ethic Committee of Huazhong Agriculture University (HZAUMO-2021-0003). Written informed consent was obtained from the owners for the participation of their animals in this study.

## Author Contributions

LZ, DZ, and JY contributed to the conception and design of the study. LZ organized the database, performed the statistical analysis, and wrote the first draft of the manuscript. LZ, MI, SK, and JY wrote the sections of the manuscript. All authors contributed to manuscript revision, read, and approved the submitted version.

## Conflict of Interest

The authors declare that the research was conducted in the absence of any commercial or financial relationships that could be construed as a potential conflict of interest.

## Publisher’s Note

All claims expressed in this article are solely those of the authors and do not necessarily represent those of their affiliated organizations, or those of the publisher, the editors and the reviewers. Any product that may be evaluated in this article, or claim that may be made by its manufacturer, is not guaranteed or endorsed by the publisher.
